# Photoreceptor preservation by FAAH inhibition in a murine model of retinitis pigmentosa

**DOI:** 10.1007/s12035-026-06038-w

**Published:** 2026-07-10

**Authors:** Camila Feitosa Magalhães, Rafael de Freitas Azevedo-Repossi, Luana de Almeida-Pereira, Millena Costa Crisóstomo, Mariana Rodrigues Pereira, Karin da Costa Calaza, Hilda Petrs-Silva, Lucianne Fragel-Madeira

**Affiliations:** 1 Laboratory of Neural Development and Regeneration, Institute of Biology, Department of Neurobiology, Universidade Federal Fluminense, Niterói, Brazil; 2 Laboratory of Nervous System Chemical Signaling Institute of Biology, Department of Neurobiology, Universidade Federal Fluminense, Niterói, Brazil; 3 Retinal Neurobiology Laboratory, Institute of Biology, Department of Neurobiology, Universidade Federal Fluminense, Niterói, Brazil; 4 Laboratory of Gene Therapy and Viral Vectors, Carlos Chagas Filho Institute of Biophysics, Universidade Federal Do Rio de Janeiro, Rio de Janeiro, Brazil

**Keywords:** Inherited retinopathy, Photoreceptor degeneration, *rd10* mice, Endocannabinoid system, Anandamide, Retinal neuroprotection

## Abstract

Retinitis pigmentosa is a hereditary neurodegenerative disease characterized by gradual photoreceptor loss, often leading to blindness. The murine model *Pde6b*^*rd10/rd10*^ (*rd10*) reproduces key features of retinitis pigmentosa (RP) and is widely used to evaluate therapeutic strategies. Anandamide is an endocannabinoid ligand degraded by fatty acid amide hydrolase (FAAH), whose levels have been shown to be increased in retinopathies. In this study, endocannabinoid signaling was pharmacologically augmented in *rd10* mice to prolong photoreceptor survival. FAAH is present both in *rd10* and C57Bl/6 retinas, with no differences in expression by qPCR or immunofluorescence analysis. To increase levels of endocannabinoid ligands URB597 (FAAH inhibitor), was administered daily by intraperitoneal injection (0.3 mg/kg), from P13 to P18 or P24. At P19, URB597 increased peripheral photoreceptor cell number by 35% and ONL thickness by 27%, with no effect in the central retina. At P25, peripheral photoreceptor number increased by 28%, although ONL thickness was unchanged. FAAH inhibition reduced TUNEL-positive cells in the peripheral retina by 50% and 53% at P19 and P25, respectively. No changes to reactive gliosis markers and microglia cells following FAAH inhibition were observed by assessing GFAP fluorescence intensity and Iba-1^+^ cell counts either in central or peripheral retina in both ages studied. Treatment with URB597 led to a 30% reduction in reactive oxygen species content at P19. Together, these data indicate a neuroprotective role of the endocannabinoid system in the context of photoreceptor degeneration in retinitis pigmentosa.

## Introduction

The retina can be affected by insults and cellular degeneration, either acquired or inherited. Proteins present in photoreceptors and pigmented epithelium are the main ones to be affected in hereditary retinopathies and to trigger vision loss [1]. Many hereditary retinopathies lead to progressive vision loss due to photoreceptor degeneration. Between those are Stargardt's disease, rod-cone dystrophy, Leber's congenital amaurosis and retinitis pigmentosa (RP) [2–4]. RP is the leading cause of blindness among hereditary retinopathies. This disease is progressive, heterogenous, neurodegenerative, with high incidence, gradually affecting photoreceptors [5–8]. Data from the RetNet network (Retinal Information Network) shows that over 100 genes are related to RP, following different types of mendelian inheritance, autosomal dominant, autosomal recessive and X-linked [9, 10]. Epidemiological data from Northern China and Denmark point to an approximate prevalence of 1:4000 people affected by RP [11, 12]. In Brazil, RP is the most prevalent pathology among patients with inherited retinal dystrophies [13]. Nyctalopia is the first clinical symptom in humans, impairing vision in low light environments, followed by visual field constriction; both caused by the initial death of rod cells. Later, central vision loss occurs when cones are affected [14]. The term “pigmentosa” refers to fundus hyperpigmentation at advanced stages, resulting from melanin deposits from the pigment epithelium that moves towards inner parts of the retina. These deposits are clinically described as bone spicules and can be observed in fundus examination [15].

There are several spontaneous animal models, which share the same mutated genes present in patients and can be used to study RP [16]. This has allowed extensive research in the area of RP and a better understanding of its underlying mechanisms. Between murine models used to study RP, there are those categorized as retinal degeneration (commonly called rd) that mimic this disease. Presently, sixteen different murine models are listed, among which ten are rd (*rd1* to *rd10*), each with its specificities in disease progression and gene mutation site [17, 18]. The *Pde6b*^*rd10/rd10*^ (*rd10*) line has an autosomal recessive inheritance pattern, observed in 60% of human patients with RP, among which, 5% carry a mutation in the phosphodiesterase gene [5]. The murine model *rd10* has a missense mutation at exon 13, substituting an arginine at position 560 to a cysteine, causing a lower activity of PDE6 in these mice. This reminiscing activity gives *rd10* a slower degeneration compared to other models, which can be divided into two phases. In the first stage, thickness of ONL decreases by approximately 80% from the beginning of the third postnatal week to the fourth week (P28). The second phase of degeneration is slower and can last up to 2 postnatal months [19]. The massive cell death in the ONL leads to anatomical [20, 21] and functional [22] remodeling of other retinal layers due to loss of synaptic inputs. Indirect disease mechanisms accelerate photoreceptor loss, such as autophagic flux dysregulation [23], reactive gliosis [24], and activation of cell death pathways [25]. Among these, excessive production of reactive oxygen species (ROS) is present in multiple models of RP as the retina is highly susceptible to oxidative damage due to its high metabolic activity, abundance of polyunsaturated fatty-acids and light exposure [26].

Endocannabinoids such as anandamide (AEA) and 2-arachidonoyl glycerol (2-AG) have been shown to act as neuroprotective agents in neurodegenerative diseases such as Alzheimer's [27], Parkinson's [28], Huntington's disease [29] and retinopathies [30–33]. Immunofluorescence analysis of endocannabinoid system components in the mouse retina demonstrated the presence of synthesis (DAGL) and degradation (MAGL; ABHD6; FAAH; NAAA) enzymes in retinal cells (retinal pigmented epithelium, photoreceptors and bipolar cells) of animals with approximately 5 weeks old [34]. In Long-Evans rats, FAAH and DAGL are expressed mainly by photoreceptors (DAGL—P1 until P60, FAAH—P7 until P60), bipolar (P7 until P60) and ganglion cells (P1 until P60), while MAGL is present in amacrine and Müller cells since P11 until P60 [35, 36].

Activation or inhibition of the endocannabinoid system in the retina can exert neuroprotective effects in a context-dependent manner [31, 37–40]. These findings highlight the complexity of the endocannabinoid system in different models. In the RP context, the cannabinoid agonist HU210 showed neuroprotective effects in the P23H rat model of autosomal dominant RP. Intraperitoneal administration of HU210 proved to be effective in preserving the outer segment of photoreceptors, as well as improving retinal functionality through electrophysiological response [41]. Another pharmacological approach is indirect activation of cannabinoid receptors by increasing its endogenous ligands. In an *in vivo* model of high intraocular pressure induced ischemia, raising endogenous levels of anandamide through intraperitoneal application of a FAAH inhibitor (URB597) preserved retinal ganglion cells [42]. Similarly, in a model of optic nerve axotomy, FAAH inhibition promoted retinal ganglion cell protection in a CB1-dependent manner [43]. Therefore, based on the presence of endocannabinoid system components and previous evidence of endocannabinoid-mediated neuroprotection in retinopathies, the present study aimed to analyze a potential neuroprotective effect of FAAH inhibition on photoreceptor degeneration in the *rd10* model of RP.

## Materials and Methods

### Materials

Drug (3'-(aminocarbonyl) [1,1'-biphenyl] −3-yl)-cyclohexylcarbamate (URB597) (cat#10,046) and polyclonal rabbit anti-FAAH (cat#101,600) were obtained from Cayman Chemicals. Paraformaldehyde (cat#158,127), poly-L-lysine (cat#P2636), Triton X-100 (cat#23,472–9), bovine serum albumin (BSA) (cat#A2153), 4′,6-diamidino-2-phenyl-indole (DAPI) (cat#D9542) and dimethyl sulfoxide (DMSO) (cat#D2650) were obtained from Sigma–Aldrich. OCT was purchased from Tissue-Tek, Sakura. Polyclonal rabbit anti-recoverin was purchased from Merck Millipore (cat#AB5585). Polyclonal rabbit anti-Iba1 was purchased from Fujifilm Wako Pure Chemical (cat#019–19741). Polyclonal rabbit anti-GFAP was obtained from Dako (cat#Z0344). Kit Click-iT Plus TUNEL Assay for In Situ Apoptosis detection (cat#C10618), TRIzol reagent (cat#15,596,026), SuperScript® III (cat#18,080,051), H2DCF-DA (cat#D399), Goat anti-Rabbit IgG secondary antibody Alexa 488 (cat#A11008) and Goat anti-Rabbit IgG secondary antibody Alexa 568 (cat#A11011), were obtained from Thermo Fisher Scientific. GoTaq qPCR Master Mix (cat#A600A) was purchased from Promega.

### Animals

All procedures involving C57Bl/6 J (wild-type) and B6.CXB1-*Pde6b*^*rd10*^/J *rd10* mice were conducted in accordance with the ARVO Statement for the Use of Animals in Ophthalmic and Vision Research. C57Bl/6 J (RRID: IMSR_JAX:000664) and *rd10* (RRID: IMSR_JAX:004297) were obtained from an in-house breeding colony originally derived from The Jackson Laboratory. Experiments were approved by the Committee for the Use of Experimental Animals of the Fluminense Federal University (Protocol No. 1464280219/2019). Animals were kept in microisolator cages in the experimental animal facility of the Department of Neurobiology, Institute of Biology, Fluminense Federal University, under a light–dark cycle of 12 h/12 h, with ad libitum access to water and food. Male and female mice were used in all experiments, without distinction. Before preparing the biological material for histological processing or RT-qPCR, animals were euthanized by isoflurane overdose followed by cervical dislocation.

### RT-qPCR

For qPCR analysis, wild-type and *rd10* retinas were used at 13, 19 and 25 postnatal ages. Total RNA was extracted from retina samples using TRIzol reagent following the manufacturer’s instructions. Subsequently, RNA was quantified using a NanoDrop 2000/2000c. For cDNA synthesis, the SuperScript III First-Strand Synthesis kit was used, following the manufacturer’s protocol. The samples of cDNA were amplified using GoTaq qPCR Master Mix and performed on RT-PCR StepOne Applied Biosystems system under the following conditions: initial denaturation at 95ºC for 2 min, followed by 40 cycles: denaturation at 95ºC for 15 s, annealing and extension at 60ºC for 1 min. Data were obtained and analyzed using StepOne software. All experiments were performed in triplicate. The relative amount of mRNA was calculated according to 2-ΔΔCt method. For this, the Ct values ​​of *Faah* were subtracted from the Ct values ​​of *Gapdh* (housekeeping gene), thus obtaining the value of ΔCt. ΔΔCt values ​​were obtained by subtracting the ΔCt mean. Finally, the values ​​found for ΔΔCt were used as a negative exponent in base 2 (2-ΔΔCt) [44]. Primer sequences used:

- *Faah* (NM_010173.5) 5’CACGCTGGTCCCCTTCTTAC3’, 5’GGCGATACATCTCAATCTCATGC3’;
- *Gapdh* (NM 001289726.1) 5’CCCTTAAGAGGGATGCTGCC3’, 5’ACTGTGCCGTTGAATTTGCC3’.

### Intraperitoneal Injections

Injections were performed in the peritoneal region with a 50-unit BD Ultrafine insulin syringe (6 mm long – 0.25 mm caliber 31G) after local asepsis with 70º GL ethyl alcohol. *rd10* mice received daily intraperitoneal (i.p) injections of FAAH enzyme inhibitor (URB597) in a fixed volume of 50 μl at a dose of 0.3 mg/kg. URB597 was reconstituted in DMSO and diluted in 0.01 M phosphate buffer (PBS). Control animals (CTR) received i.p injections of DMSO:PBS. Applications started during the period that covered the eye opening – mostly at 13 postnatal days – until the ages of 18 and 24 postnatal days. Eyes were harvested 24 h after the last injection (P19 and P25).

### Tissue Histology

After different survival times, wild-type and *rd10* mice were euthanized as previously mentioned. The eyes were removed by enucleation with curved surgical tweezers and placed in a petri dish containing 4% paraformaldehyde in 0.01 M PBS pH 7.4 for 5 min for a brief fixation. Then, 2 Swiss tweezers and an iridectomy scissors were used to remove cornea and lens in 0.01 M PBS. Following this, eyecups were fixed in 4% paraformaldehyde for 55 min at room temperature. Afterwards, the eyes were washed 3 times with a 0.01 M PBS and kept at 4ºC. For tissue preservation, a sucrose gradient was used (10%, 20% and 30%) diluted in 0.01 M PBS for 24 h at 4º C. Eyes were prepared for cryosection by embedding in Optimum Cutting Temperature medium (OCT), oriented under a stereoscopic microscope and frozen with liquid nitrogen. Frozen eyes were cut in a Leica CM1850 cryostat at a thickness of 10 µm and collected in glass slides pre-treated with poly-L-lysine (200 µg/ml) [45]. Slides with retinal sections were kept at −20º C until further analysis.

### Immunofluorescence

Retinal sections were incubated in 0.5% Triton X-100 for 15 min at room temperature to permeabilize cell membranes and then washed twice with PBS for 5 min. Blocking of nonspecific sites was performed with 1% bovine serum albumin (BSA) at room temperature for 30 min. After removing BSA, sections were incubated overnight with primary antibodies: polyclonal rabbit anti-recoverin (1:1500) or polyclonal rabbit anti-IBA1 (1:600) or polyclonal rabbit anti-GFAP (1:200) or polyclonal rabbit anti-FAAH (1:25) diluted in 1% BSA and kept at 4ºC in a refrigerator. Slides were washed twice with PBS (5 min each) followed by incubation with fluorescent secondary goat anti-rabbit antibody Alexa Fluor 488 or 568 (1:200) diluted in 1% BSA for 2 h at room temperature and protected from light. Slides were washed twice with PBS for 5 min and, consecutively, 4,6'-diamino-2-phenyl-indole (DAPI) 1 μg/mL was applied to the sections for 3 min for cell nuclei stain. One last wash was done with PBS. Following this, coverslips were mounted on the slides with 4% n-propyl-gallate: 80% glycerol in PBS, followed by sealing. Photomicrographs were obtained on a Leica DM2500 epifluorescence microscope using the LasV 3.7 program. Negative controls were performed by omitting the primary antibody during the incubation step. During analysis under the microscope these were used as a baseline to remove the background staining of the images. All photomicrographs were captured with the same magnification (40x).

### Terminal Deoxynucleotidyl Transferase-Mediated dUTP Nick-end Labeling (TUNEL) Assay

DNA fragmentation analysis was performed using the Click-iT Plus TUNEL Assay kit (ThermoFisher Scientific) following the manufacturer's instructions. Briefly, slides with retinal sections were permeabilized with 0.5% Triton X-100 for 15 min at room temperature and then washed twice with deionized water for 5 min. Then, TdT (Terminal deoxynucleotidyl Transferase) reaction buffer was added for 10 min at 37 °C. After this step, TdT reaction buffer excess was removed and TdT reaction mixture was added (composed of TdT reaction buffer, EdUTP and TdT enzyme) for 60 min at 37ºC. The TdT reaction mixture causes the TdT enzyme to incorporate EdUTP into the DNA fragments. Slides were washed twice with 3% BSA for 5 min and incubated with the Click-iT® Plus TUNEL reaction cocktail for 30 min at 37ºC. From this step onward, slides were protected from light. This cocktail has fluorophores that bind to EdUTP, allowing identification of cells with fragmented DNA. Afterwards, slides were washed once with 3% BSA for 5 min, incubated with 1 μg/ml DAPI for 3 min, washed with PBS for 5 min, mounted with n-propyl gallate and sealed. Observation was performed using a Leica DM2500 epifluorescence microscope and photomicrographs captured by LasV 3.7 program at 40 × magnification.

### Cell Quantification and Outer Nuclear Layer Thickness

Due to differences during degeneration between peripheral and central retinal areas in *rd10* animals, four different fields from each eye were photographed, totaling eight fields per experimental point for recoverin and TUNEL positive cell counting. Central fields were considered as areas adjacent to the optic nerve (100 µm from the optic nerve) and peripheral fields, 100 µm from the outermost end of the retina. Recoverin or TUNEL positive cells were counted manually by an individual blinded to the treatment groups in photomicrographs containing 120 µm of retinal length using ImageJ v1.50i software. All cells immunolabeled for recoverin in the ONL that showed DAPI staining were considered recoverin positive cells. ONL thickness was measured using Image J v1.50i program by averaging 3 different regions of each photomicrograph. Microglial cells are irregularly present along the retina, and their cell bodies can be in different viewing planes. Therefore, quantification of Iba-1 positive cells was performed directly under the microscope at 400 × magnification. The number of Iba-1-labeled microglial cells was quantified both in the total retina (nuclear and plexiform layers) and separately in the ONL, along the entire length of the retina.

### Fluorescence Intensity Quantification

Analysis of reactive gliosis was performed by measuring fluorescence intensity of sections labeled with anti-GFAP. All GFAP immunohistochemistry assays were performed on the same day, using the same parameters (exposure, gain and gamma) for the Alexa 568 (red) channel. Photomicrographs were obtained in a Leica DM2500 microscope, using the LasV 3.7 program, at 40 × magnification, and analyzed using Adobe Photoshop CC 2015. Labeling of glial filaments of a region corresponding to 120 µm of the retina was selected and, using the histogram tool, average pixel intensity in the red channel was obtained.

### Quantification of ROS in the Retinas Using H_2_DCF-DA

H_2_DCF-DA is a fluorescent probe that readily oxidizes in the presence of ROS, emitting fluorescence in the 522 nm wavelength, thus it is widely used as a probe to ROS levels in the cells. The protocol was based on Osada et al. 2017 [46], with some modifications. Following treatment with URB597 0.3 mg/kg from eye opening until P18, *rd10* mice were euthanized at P19 as previously described. Eyes were enucleated immediately after euthanasia and placed in 0.01 M PBS. Using style 5 fine point tweezers, an incision was made into the cornea, and the eye was opened by pulling the cornea and sclera apart in opposite directions. The retina was gently peeled from the sclera and both lens and ciliary body were removed. Extra care was taken as to not damage any retinal tissue. This process was performed quickly and with as little light as possible so as not to influence the results. Afterwards, a pool of two retinas was flash frozen on liquid nitrogen, immediately placed onto 100 μL of PBS and vigorously disrupted using a pestle. To ensure lysis, samples were vortexed at 2800 rpm for 30 s using a Biomixer QL-901 vortex mixer. Following this, H_2_DCF-DA was added to the samples to a concentration of 50 μM and incubated at 37ºC for 1 h. Afterwards, cells were pelleted by centrifugation at 6400 RPM for 5 min in a Kasvi K14-0602 centrifuge at 4ºC. Pellets were washed twice with 0.01 M PBS and resuspended in PBS by vortexing briefly. Resuspended pellets were transferred to a non-treated black 96-well plate, and fluorescence intensity was measured using a BioTek Synergy H1 plate reader with excitation at 493 nm and emission at 522 nm. Negative control blanks were prepared by performing the same procedure in samples lacking retinal tissue.

### Statistical Analysis

All statistical analysis was conducted using the GraphPad Prism software, version 10.6.1 (GraphPad Software, Inc., San Diego, CA). All samples with three or more independent experiments were accepted in the Shapiro–Wilk normality test and were presented as the mean ± standard error of the mean (SEM). Unpaired Student’s t-test with Welch correction was used for statistical analysis of most experiments. One-way ANOVA followed by Dunnett’s post hoc test was used to compare relative FAAH mRNA expression across *rd10* ages. P values ​​less than 0.05 were considered statistically significant.

## Results

### Photoreceptor Degeneration Time-Course Analysis in *rd10* Animals

**Fig. 1:**
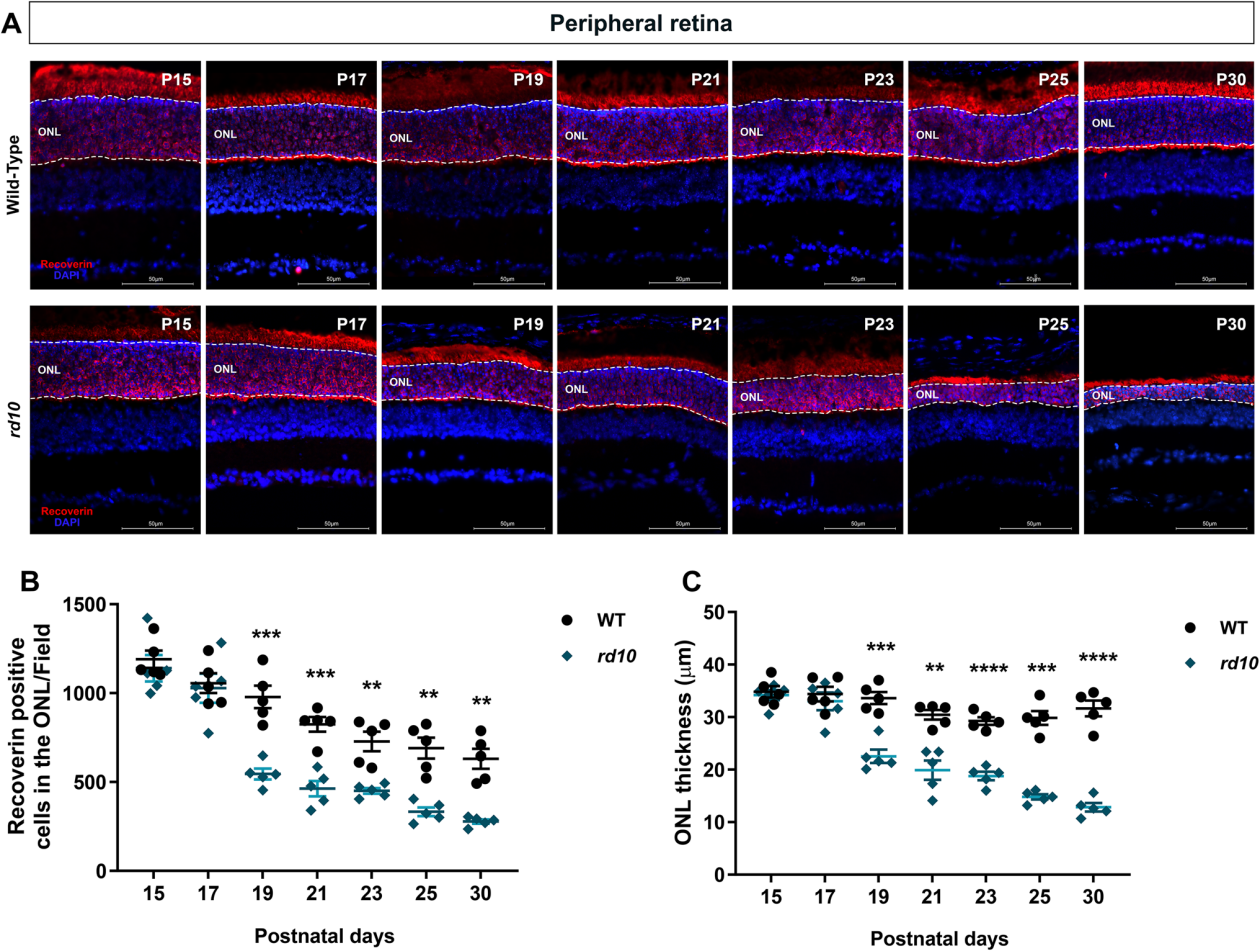
Comparison between strains demonstrated onset of photoreceptor degeneration in retinal periphery at P19 in *rd10* mice. Histological sections of the retina’s peripheral area immunostained for recoverin obtained from WT and *rd10* animals. **A** Photomicrographs compare photoreceptor cells (red) at different postnatal ages (15, 17, 19, 21, 23, 25 and 30), and ONL thickness (white dashed lines). Nuclei were counterstained with DAPI (blue). **B** Analysis of peripheral fields evidenced a cellular reduction from P19 to P30 on *rd10* compared to WT. Black circles represent WT animals and blue diamonds *rd10*. **C** Quantification of ONL thickness in peripheral fields shows retinal thinning starting at P19 in *rd10* animals. ONL—Outer nuclear layer. Calibration bar = 50 μm. Retinal field = 120 μm. Results represent the mean ± SEM of five independent experiments. Each age was analyzed independently using an unpaired Student’s *t*-test with Welch’s correction. **p < 0.01; ***p < 0.001; ****p < 0.0001

**Fig. 2:**
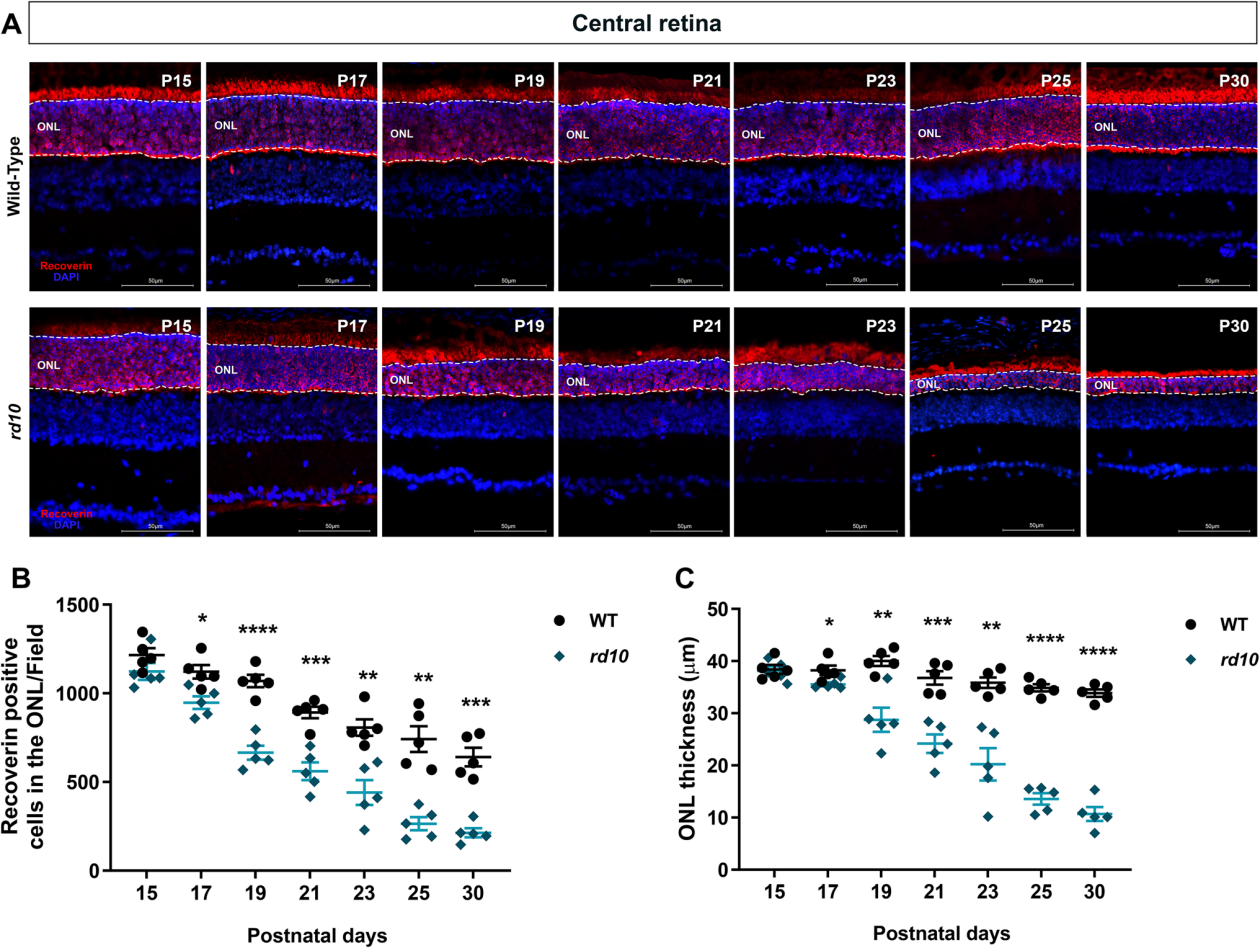
Comparison between strains demonstrated onset of photoreceptor in retinal center at P17 in *rd10* mice. Histological sections of the retina’s central area immunostained for recoverin obtained from WT and *rd10* animals. **A** Photomicrographs compare photoreceptor cells (red) at different postnatal ages (15, 17, 19, 21, 23, 25 and 30), and ONL thickness (white dashed lines). Nuclei were counterstained with DAPI (blue). **B** Analysis of central areas demonstrated loss of photoreceptors cells at P17, with a center-periphery pattern. Reduction in photoreceptor numbers continues until P30 in *rd10* animals. Black circles represent WT animals and blue diamonds *rd10*. **C** In the central retina, ONL thinning is present at P17, following the same pattern of photoreceptor decrease. ONL—Outer nuclear layer. Calibration bar = 50 μm. Retinal field = 120 μm. Results represent the mean ± SEM of five independent experiments. Each age was analyzed independently using an unpaired Student’s *t*-test with Welch’s correction. *p < 0.05; **p < 0.01; ***p < 0.001; ****p < 0.0001

The *rd10* model has a pattern of degeneration in which cells degenerate first in the central region and then in the peripheral region of ​​the retina [22]. Therefore, our first analysis was to evaluate the number of photoreceptors present in the retina of *rd10* animals and compare it to a healthy retina (WT). Retinal degeneration was analyzed from P15, when all retinal cell types are differentiated, to P30, when retinal maturation is achieved [47, 48]. Recoverin immunolabeling was used to quantify cones and rods and to delineate the ONL. To examine temporal differences in photoreceptor death between strains, peripheral (Fig. 1a) and central (Fig. 2a) regions of the retina were analyzed separately. Quantification of recoverin-positive cells demonstrated that, in peripheral areas, P15 (WT: 1190 ± 49; *rd10*: 1140 ± 74; *p* = 0.5896) and P17 (WT: 1056 ± 56; *rd10*: 1028 ± 82; *p* = 0.7875) had no differences in the number of photoreceptors. However, at P19 (WT: 979 ± 63; *rd10*: 545 ± 31; *p* = 0.0010) the amount of photoreceptors decreased in *rd10*. This peripheral degeneration persisted until the 30th postnatal day: P21 (WT: 824 ± 41; *rd10*: 463 ± 43; *p* = 0.0003), P23 (WT: 728 ± 55; *rd10*: 450 ± 16; *p* = 0.0056), P25 (WT: 690 ± 59; *rd10*: 333 ± 25; *p* = 0.0020) and P30 (WT: 631 ± 56; *rd10*: 278 ± 12; *p* = 0.0027) (Fig. 1b). To corroborate findings related to photoreceptor loss, ONL thickness was also quantified. At P15 (WT: 34.8 ± 1.1 μm; *rd10*: 34.2 ± 1.0 μm; *p* = 0.6713) and P17 (WT: 34.4 ± 1.4 μm; *rd10*: 33.0 ± 1.7 μm; *p* = 0.5480), no differences in ONL thickness were observed between WT and *rd10* animals in the peripheral retina. However, starting at P19, ONL thickness progressively decreases in the rd10 strain until P30: P19 (WT: 33.6 ± 1.1 μm; *rd10*: 22.5 ± 1.3 μm; *p* = 0.0002), P21 (WT: 30.4 ± 0.9 μm; *rd10*: 19.9 ± 1.8 μm; *p* = 0.0022), P23 (WT: 29.3 ± 0.7 μm; *rd10*: 18.8 ± 0.8 μm; *p* < 0.0001), P25 (WT: 29.8 ± 1.3 μm; *rd10*: 14.8 ± 0.5 μm; *p* = 0.0001) and P30 (WT: 31.6 ± 1.5 μm; *rd10*: 12.8 ± 0.8 μm; *p* < 0.0001) (Fig. 1c). In the central retina, a reduction in photoreceptor number is not detected at P15 (WT: 1216 ± 39; *rd10*: 1123 ± 47; *p* = 0.1663). A decline in photoreceptor number was observed at P17 (WT: 1122 ± 38; rd10: 947 ± 35; *p* = 0.0103), becoming more pronounced at later ages: P19 (WT: 1069 ± 35; *rd10*: 665 ± 39; *p* < 0.0001); P21 (WT: 892 ± 33; *rd10*: 561 ± 50; *p* = 0.0009); P23 (WT: 807 ± 46; *rd10*: 440 ± 70; *p* = 0.0033); P25 (WT: 742 ± 73; *rd10*: 265 ± 37; *p* = 0.0012) and P30 (WT: 641 ± 52; *rd10*: 214 ± 26; *p* = 0.0004) (Fig. 2b). At P15, ONL thickness in the central retina remained unchanged (WT: 38.4 ± 0.9 μm; *rd10*: 38.1 ± 0.9 μm; *p* = 0.8253). A reduction in ONL thickness was observed in rd10 animals at P17 compared with WT animals (WT: 38.2 ± 0.9 μm; *rd10*: 35.5 ± 0.4 μm; p = 0.0380). During the third to fourth postnatal weeks, ONL thinning became more apparent in the rd10 retina: P19 (WT: 40.0 ± 1.0 μm; *rd10*: 28.7 ± 2.3 μm; *p* = 0.0053), P21 (WT: 36.8 ± 1.3 μm; *rd10*: 24.2 ± 1.8 μm; *p* = 0.0006), P23 (WT: 35.9 ± 1.0 μm; *rd10*: 20.2 ± 3.1 μm; *p* = 0.0054), P25 (WT: 34.9 ± 0.7 μm; *rd10*: 13.6 ± 1.1 μm; *p* < 0.0001) and P30 (WT: 33.9 ± 0.7 μm; *rd10*: 10.7 ± 1.3 μm; *p* < 0.0001) (Fig. 2c). Based on these results, it is possible to observe a two-day delay in the temporal course of degeneration between the central and peripheral areas of the retina, with degeneration initiating at P17 in the central retina. Photoreceptor degeneration progresses throughout the retina until P30, resulting in few remaining cells in the ONL.

### FAAH Expression in *rd10* Mice Retinas

**Fig. 3:**
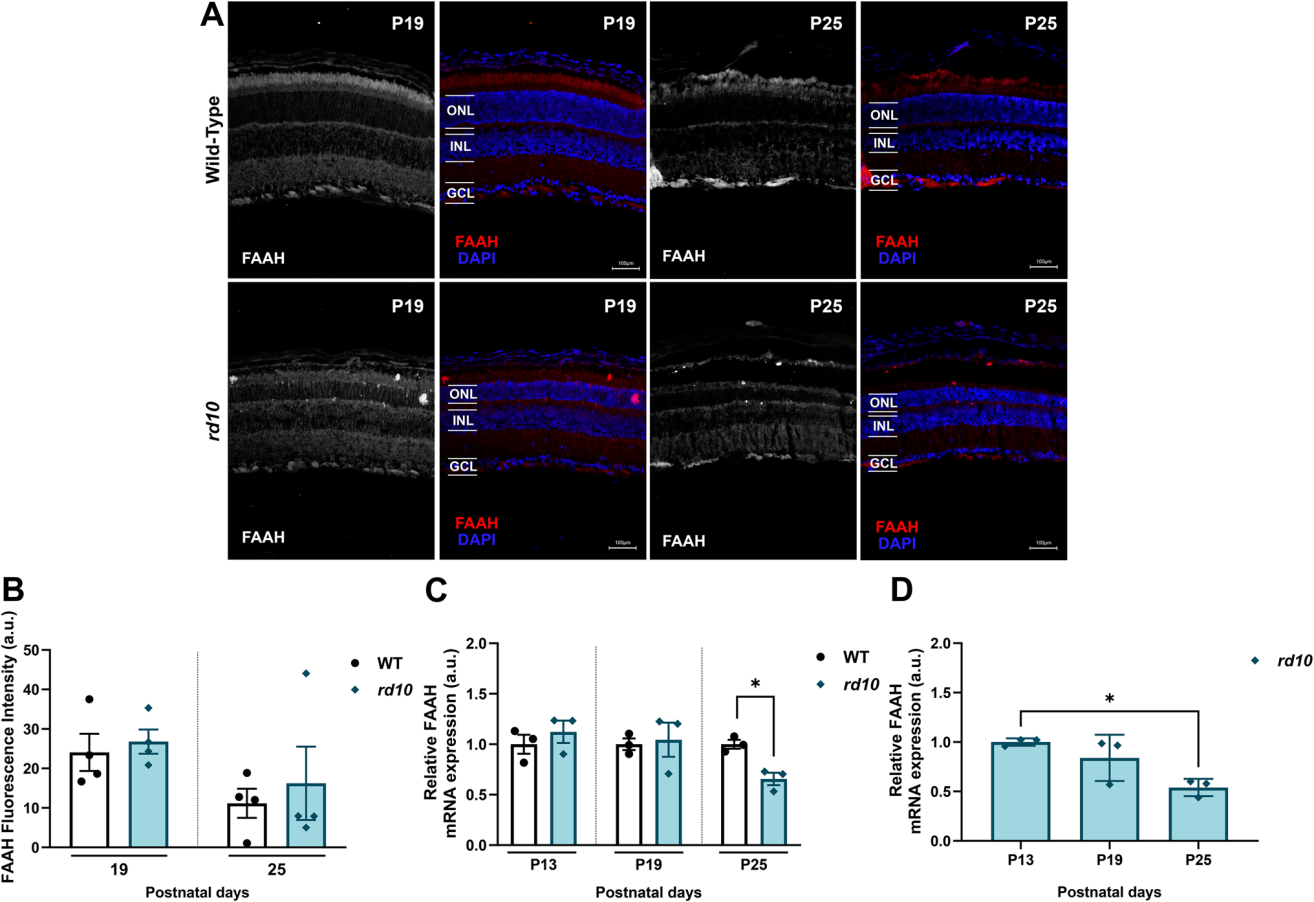
Expression and quantification of FAAH in the murine retina. **A** Retinas immunolabeled via immunofluorescence against FAAH showed a similar pattern of labeling between WT and *rd10* animals throughout the retina at ages P19 and P25. FAAH was diffusely present in all retinal layers. **B** Fluorescence intensity analysis has shown equal levels of FAAH between WT and *rd10* mice in both ages analyzed P19 and P25. **C** Comparison of FAAH mRNA expression among animals of the same age revealed no significant difference between P13 and P19; however, at P25, rd10 animals exhibited lower expression levels. **D** In rd10 animals, FAAH mRNA expression decreased at P25 compared to P13. Black circles represent WT animals and blue diamonds *rd10*. au = arbitrary units. Results represent the mean ± SEM of three independent experiments. Each age was analyzed independently using an unpaired Student’s *t*-test with Welch’s correction, except D, which was one-way ANOVA. *p < 0.05

FAAH is the main anandamide degrading enzyme and has been described in the retina of different mammals, including adult mice [49]. FAAH was expressed throughout the retina in young mice. Interestingly, both WT and *rd10* showed a similar pattern of FAAH distribution in P19 and P25 retinas (Fig. 3a). Quantification of FAAH fluorescence intensity revealed no differences between WT and *rd10* retinas at P19 (WT: 24.1 ± 4.7; *rd10*: 26.8 ± 3.1; p = 0.6469) and P25 (WT: 11.2 ± 3.7; *rd10*: 16.2 ± 9.3; p = 0.6405) (Fig. 3b). WT and *rd10* exhibit the same levels of FAAH enzyme mRNA expression in the retina of P13 (WT: 1.000 ± 0.094; *rd10*: 1.123 ± 0.110; p = 0.4452) and P19 (WT: 1.000 ± 0.0578; *rd10*: 1.045 ± 0.1691; p = 0.8208). At P25, rd10 retinas exhibited reduced FAAH mRNA levels (WT: 1.000 ± 0.0434; *rd10*: 0.6553 ± 0.0615; p = 0.0131) (Fig. 3c). Similarly, analysis across rd10 ages demonstrated no difference at P19 (0.84 ± 0.1356; p = 0.5576) and a lower mRNA expression at P25 (0.54 ± 0.0508; p = 0.0311) compared with P13 (1.000 ± 0.0215) (Fig. 3d). Thus, these results demonstrate for the first time the presence of FAAH in both healthy and degenerating mice retinas during the first four postnatal weeks.

### FAAH Selective Inhibitor, URB597, Slowed Photoreceptor Degeneration in *rd10* Animals

**Fig. 4:**
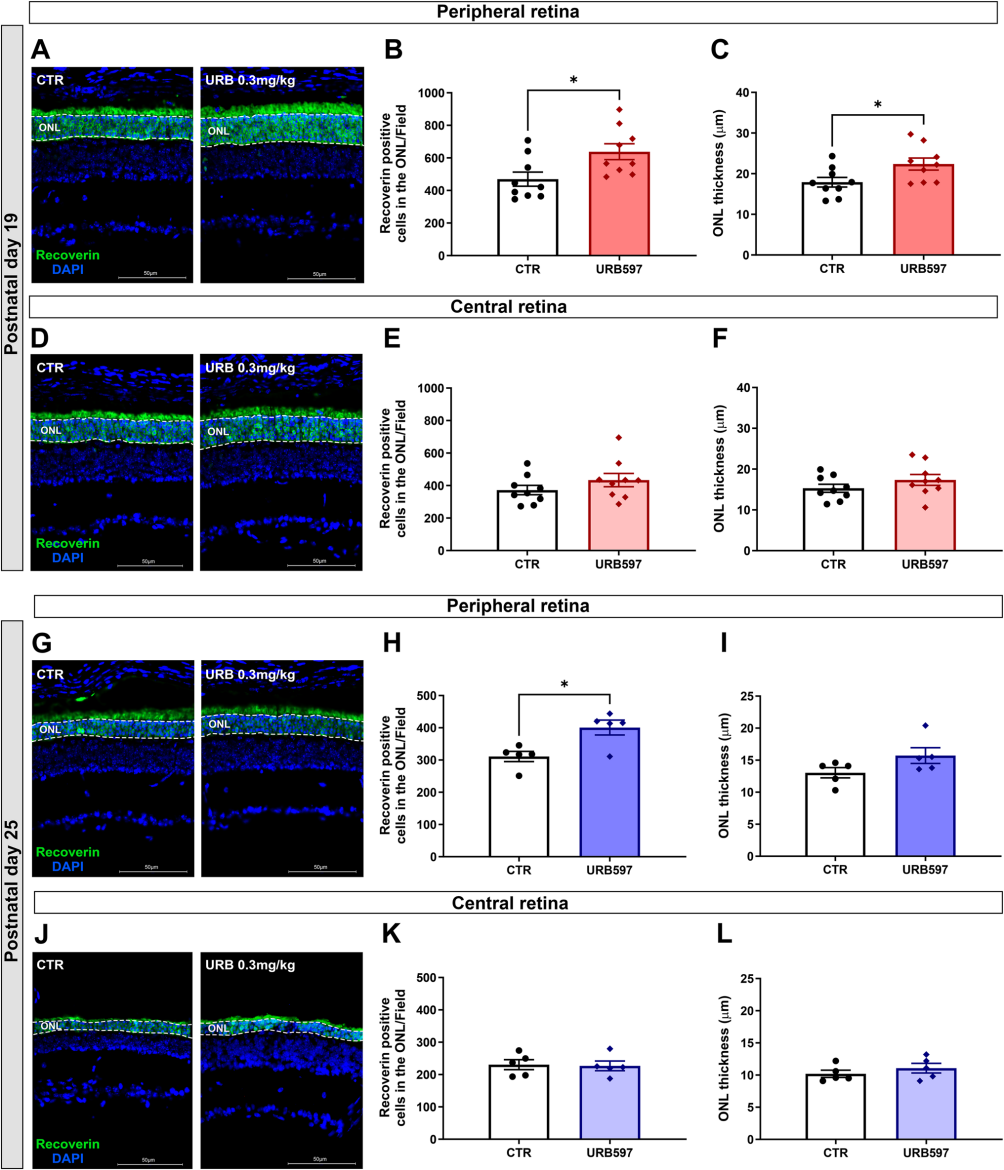
Subchronic treatment with the FAAH inhibitor URB597 preserves photoreceptor number and ONL thickness in *rd10* mice. Representative histological retinal sections immunostained for recoverin (green) are shown. Daily administration of URB597 preserved photoreceptor number and ONL thickness at both ages analyzed. **A**–**C** Quantification of recoverin-positive cells and ONL thickness demonstrated that FAAH inhibition promoted photoreceptor preservation in the peripheral retina, accompanied by increased ONL thickness. D–F. No differences were detected in the central retina at P19 for either photoreceptor number or ONL thickness. **G**–**I** At P25, treatment increased photoreceptor number in the peripheral retina without significantly affecting ONL thickness. **J**–**L** Analysis of the central retina at P25 revealed no differences between control and treated groups in photoreceptor number or ONL thickness. Scale bar = 50 μm; retinal field = 120 μm. Data are expressed as the mean ± SEM from nine independent experiments (P19) and five independent experiments (P25). *p < 0.05

To characterize the effects of endocannabinoid upregulation on photoreceptor degeneration, a selective FAAH inhibitor (URB597) was administered intraperitoneally. At P19, *rd10* animals treated with URB597 0.3 mg/kg showed a 35% increase in the number of recoverin positive cells in the ONL compared to controls (CTR: 470 ± 43; URB597: 638 ± 48; p = 0.0205) (Fig. 4a, b). Corroborating the observed increase in photoreceptor number, ONL thickness of peripheral retinas was also increased by 27% in treated *rd10* mice (CTR: 17.9 ± 1.1 μm; URB597: 22.3 ± 1.5 μm; p = 0.0326) (Fig. 4c). This neuroprotective effect was not observed in central regions of the retina (CTR: 373 ± 28; URB597: 434 ± 40; p = 0.2357), likewise the ONL thickness remained similar in both groups (CTR: 15.3 ± 1.0 μm; URB597: 17.3 ± 1.3 μm; p = 0.2394) (Fig. 4d, e, f). To determine whether the neuroprotective effect observed at P19 persisted during the slower phase of degeneration, the treatment window was extended until P25. Daily i.p treatment with URB597 increased the number of recoverin positive cells in the peripheral region of the *rd10* retina by 28% (CTR: 311 ± 15; URB597: 400 ± 23; p = 0.0149) (Fig. 4g, h). As shown by P19, at P25 the number of photoreceptors in the central retina was not different between control and treated animals (CTR: 230 ± 15; URB597: 227 ± 15; p = 0.8708) (Fig. 4j, k). ONL thickness did not change in both regions studied (Periphery—CTR: 13.0 ± 0.8 μm; URB597: 15.7 ± 1.2 μm; p = 0.1122; Central—CTR: 10.2 ± 0.5 μm; URB597: 11.0 ± 0.7 μm; p = 0.3770) (Fig. 4i, l). Taken together, these data suggest that FAAH inhibition can, at least in part, delay photoreceptor degeneration of peripheral areas.

### FAAH Enzyme Blockade Decreased Cell Death in the Outer Nuclear Layer of *rd10* Mice

**Fig. 5:**
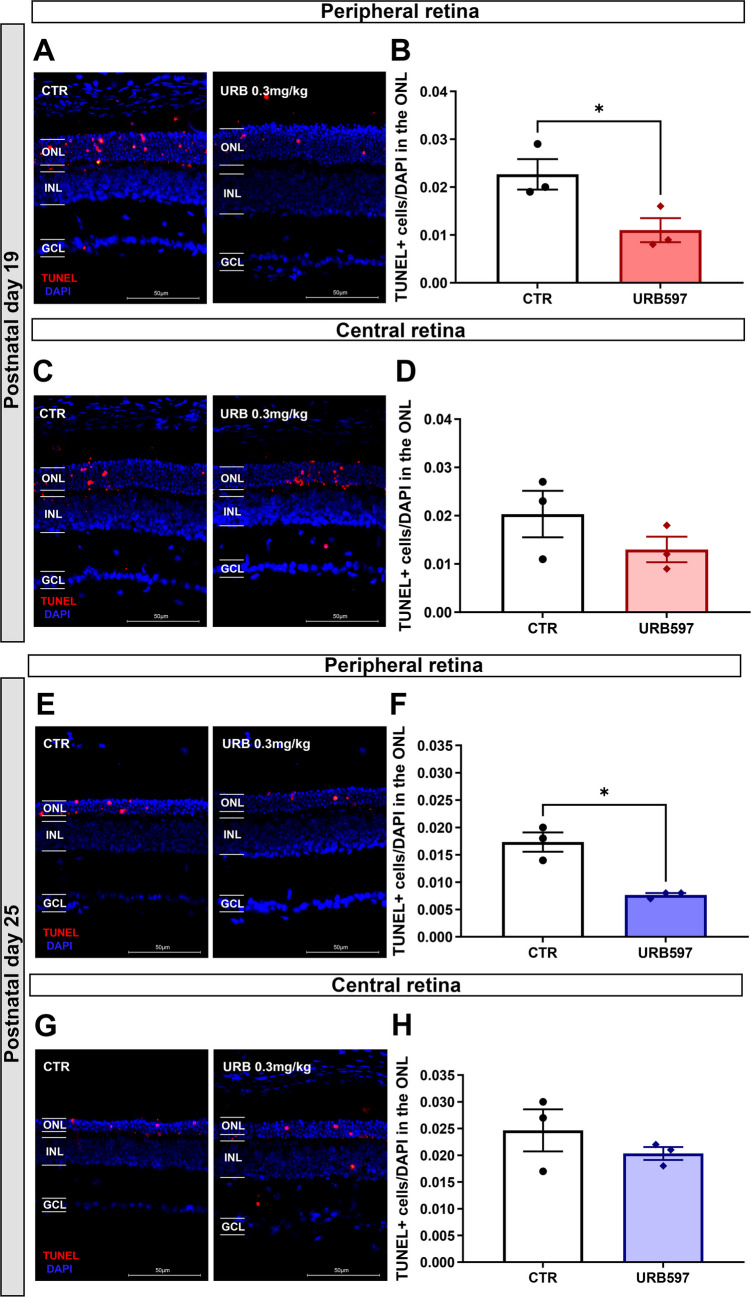
URB597 treatment decreases cell death at the peripheral region of the retina in rd10 mice at P19 and P25. Daily intraperitoneal treatment with URB597 decreased the number of TUNEL labeled dying cells (red) in the ONL at the peripheral region of both P19 and P25 *rd10* mice. **A**-**B** On the periphery of the retina, quantification of TUNEL + cells in the ONL of URB597 treated mice at P19 showed a decrease in cell death. **C**-**D** However, no effect could be observed on the number of dying cells in the center. **E**–**F** Protection of the peripheral region was also observed at P25 in treated animals compared to controls. **G**-**H** Similar to P19, the protective effect was restricted to the peripheral region of the retina, with no differences between treated and control groups in the center of the retina. ONL—Outer nuclear layer. Calibration bar = 50 μm. Retinal field = 120 μm. Results represent the mean ± SEM of nine independent experiments (P19) and five independent experiments (P25). *p < 0.05

Death of photoreceptor cells in *rd* models is complex, with evidence indicating the involvement of classical apoptotic mediators, as well as non-apoptotic cell death pathways [50, 51]. Since i.p treatment with URB597 protected photoreceptors in the peripheral retina, the present study evaluated whether this effect was associated with reduced cell death. To address this question, a TUNEL assay was performed to assess the presence of dying cells in the ONL (Fig. 5). At P19, animals treated with URB597 (0.3 mg/kg) exhibited approximately 50% fewer dying cells in the ONL of the peripheral retina compared with controls (CTR: 0.023 ± 0.003; URB597: 0.011 ± 0.002; p = 0.0480) (Fig. 5a, b), whereas no difference was observed in the ONL of the central retina (CTR: 0.020 ± 0.005; URB597: 0.013 ± 0.003; p = 0.2708) (Fig. 5c, d). This neuroprotective effect was maintained up to P25. At this age, the number of TUNEL-positive photoreceptors in the peripheral retina of URB597-treated animals was approximately 53% lower than in control animals (CTR: 0.017 ± 0.002; URB597: 0.008 ± 0.0003; p = 0.0282) (Fig. 5e, f). In the central retina, no change was detected in the number of dying photoreceptors (CTR: 0.025 ± 0.004; URB597: 0.020 ± 0.001; p = 0.3871) (Fig. 5g, h). Together, these data demonstrate that FAAH inhibition exerts a neuroprotective effect on the peripheral retina of *rd10* mice by reducing the number of dying cells in the ONL.

### Pharmacological Intervention With a FAAH Inhibitor Did Not Change Reactive Gliosis and Microglial Cell Distribution

**Fig. 6:**
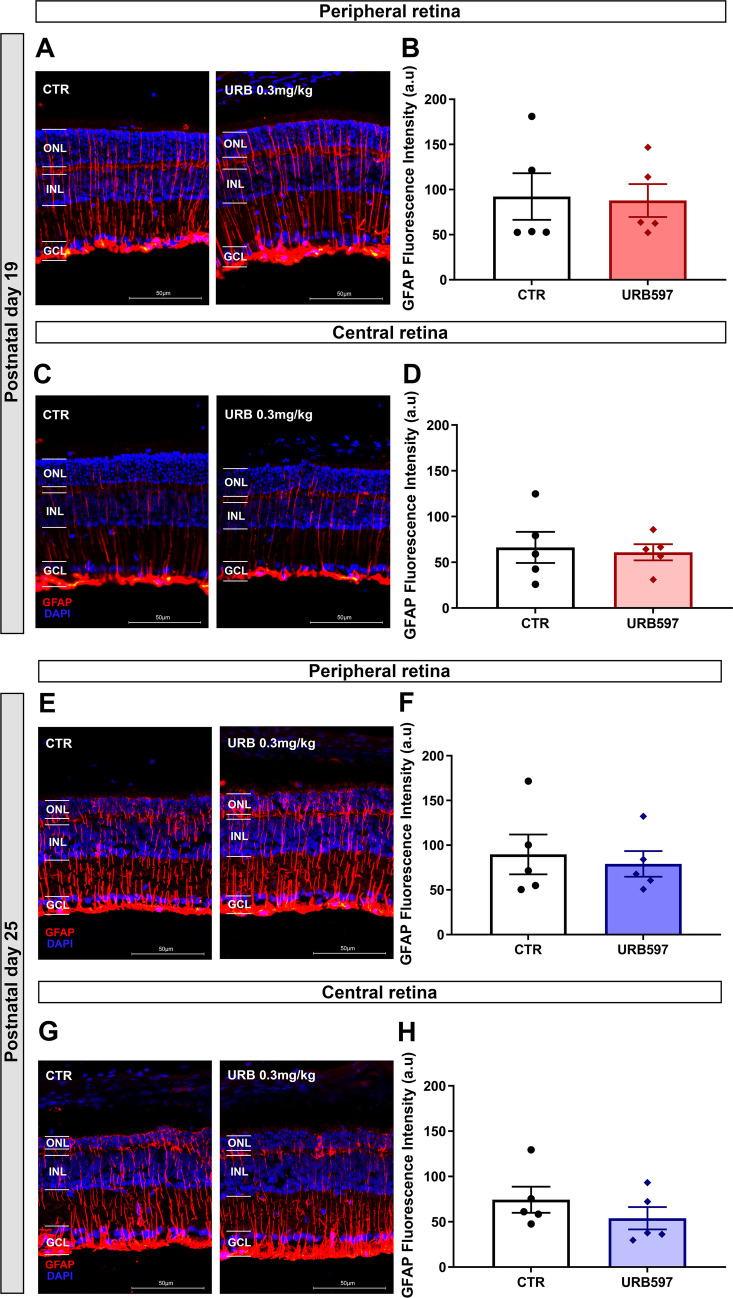
Müller Glia reactivity is not affected by FAAH inhibition at P19 and P25. Reactivity of the Müller glia was evaluated by GFAP fluorescence intensity analysis on immunolabeled retinal slices. GFAP expression is unchanged between URB597 treated and control mice. **A**-**B** Comparing GFAP labeling intensity, no differences were observed at P19 in the peripheral region. **C**-**D** Likewise, GFAP levels between both groups remained similar in the center of the retina. **E**–**F** Similarly, at P25 no differences were observed in the peripheral region. **G**-**H** Furthermore, GFAP intensity remained unchanged in the central region at P25. ONL: Outer nuclear layer; INL: Inner nuclear layer, GCL: ganglion cell layer. Calibration bar = 50 μm. Results represent the mean ± SEM of four independent experiments

**Fig. 7:**
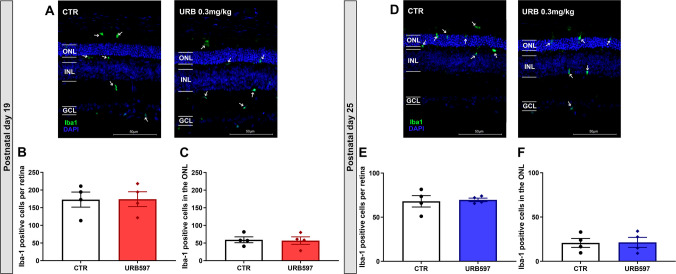
Microglial distribution in histological retinal sections after subchronic treatment with URB597 at P19 and P25 animals. Microglia cells were detected with Iba-1 (green) and are present in all layers of the retina (white arrows). **A**-**C** Upon analyzing microglia distribution in *rd10* mice treated with URB597 compared to control groups, data on either the overall number of microglia or the number of microglia in the ONL remained equal to controls. **D**-**F** Similar results were observed at P25, with the number of microglia in all retinal layers and microglia in the ONL on the same levels as controls. ONL: Outer nuclear layer; INL: Inner nuclear layer, GCL: ganglion cell layer. Calibration bar = 50 μm. Results represent the mean ± SEM of four independent experiments

Considering the reduction in degenerating cells in the ONL following URB597 treatment, the potential involvement of retinal glial cells (Müller glia and microglia) in this neuroprotective effect was investigated. *rd10* mice exhibited strong filamentous GFAP immunostaining, going from the beginning of ONL to the GCL at P19 (Fig. 6a, c) and at P25 (Fig. 6e, g). At P19, FAAH inhibition did not modify GFAP expression in either the peripheral (CTR: 92.2 ± 25.8; URB597: 87.8 ± 18.2; p = 0.8936) or central retina (CTR: 66.2 ± 17; URB597: 60.8 ± 8.8; p = 0.7890) (Fig. 6b, d). Analysis of the peripheral area at P25 did not reveal changes in the Müller glial response (CTR: 89.7 ± 22.2; URB597: 79.0 ± 14.3; p = 0.7002), nor in the central retina (CTR: 74.3 ± 14.4; URB597: 53.8 ± 12.3, p = 0.3120) (Fig. 6f, h). This does not guarantee that the treatment lacks an effect on glial responses, as endocannabinoids can modulate other glial cells, such as microglia. Therefore, Iba-1^+^ cells were analyzed along the entire retina. All Iba-1^+^ cell bodies were quantified, and a heterogeneous microglial distribution and morphology were observed in both groups. Microglial cells were detected in the plexiform layers of the retina, with additional cells infiltrating the nuclear layers (Fig. 7a, d, white arrows). At both P19 and P25, in both CTR and URB597-treated retinas, although most microglial cells were concentrated in the ONL, some cells were also observed in the OPL, INL, and GCL. The number of microglia present in the photoreceptor layer was unchanged between treated and control animals at P19 (CTR: 59 ± 8; URB597: 57 ± 10; p = 0.8737) or P25 (CTR: 21 ± 5; URB597: 21 ± 6; p = 0.9373) (Fig. 7c, f). Likewise, the total amount of microglial cells did not change when accounting for all retinal layers (P19—CTR: 173 ± 21; URB597: 174 ± 21; p = 0.9680. P25—CTR: 68 ± 6; URB597: 69 ± 2; p = 0.8241) (Fig. 7b, e).

### Inhibition of FAAH Enzyme Lowered Reactive Oxygen Species Levels in *rd10* Retinas

**Fig. 8:**
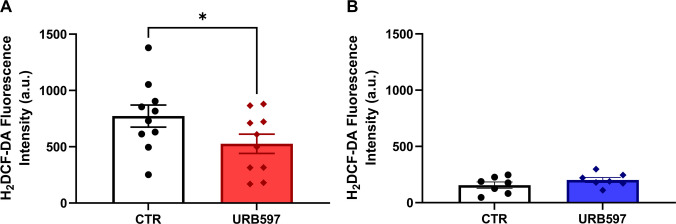
Daily FAAH inhibitor treatment lowers oxidative stress in *rd10* retinas. **A** Fluorescence analysis of the H_2_DCF-DA probe in whole retinal extracts at P19 shows that URB597 treated animals displayed lower levels of ROS compared to controls. **B** No change in fluorescence intensity of the retinas was observed at P25 between groups. Results represent the mean ± SEM of ten and seven independent experiments, respectively. *p < 0.05

Reactive oxygen species play an important role in accelerating photoreceptor death in both animal models of RP and patients [52]. Based on this evidence, the present study evaluated whether upregulation of cannabinoid signaling reduces ROS levels in treated retinas. H2DCF-DA, a probe used as a general indicator of intracellular ROS, was employed to determine whether FAAH inhibition affected ROS production in *rd10* retinas. A 30% reduction in fluorescence intensity was observed in retinas of *rd10* mice treated until P19 compared with controls (CTR: 772.7 ± 98.23; URB597: 526.4 ± 85.32; p = 0.0374) (Fig. 8a). At P25, fluorescence intensity did not differ between control and treated groups (CTR: 156.0 ± 28.05; URB597: 202.1 ± 22.61; p = 0.1131) (Fig. 8b). Notably, fluorescence intensity at P25 was lower than at P19, suggesting reduced ROS levels at a more advanced stage of retinal degeneration. Minimal fluorescence was detected in negative control blanks (CTR NEG: 3.0 ± 1.751; data not shown). Therefore, reduced retinal ROS levels may be, at least in part, responsible for the protective effects associated with FAAH inhibition.

## Discussion

**Fig. 9:**
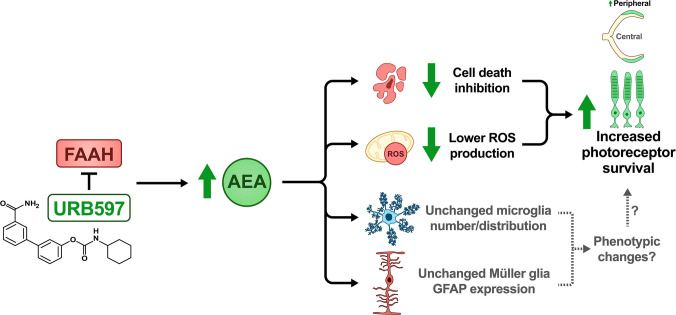
Illustrated overview summarizing possible neuroprotective mechanisms in peripheral photoreceptors mediated by FAAH inhibition. URB597 pharmacologically inhibits FAAH and may increase AEA availability in the retina. Treatment of rd10 mice reduced cell death and oxidative stress, as evidenced by a lower number of TUNEL + cells and decreased ROS production, respectively. Although no detectable changes were observed in Müller glia and microglial cell count parameters, potential phenotypic and functional contributions to retinal preservation cannot be ruled out. Together, these data indicate that endocannabinoid system modulation influences the progression of retinal degeneration, particularly in the peripheral retina, in a murine model of RP

Research exploring components of the endocannabinoid system as therapeutic agents for degenerative retinopathies has introduced a novel avenue for intervention aimed at preventing cell death. The present study demonstrates, for the first time, the presence of the FAAH enzyme in the retina of a murine model of RP and provides evidence that modulation of the endocannabinoid system can influence disease progression by delaying photoreceptor degeneration. By reducing reactive oxygen species levels and photoreceptor apoptosis, FAAH inhibition emerges as a potential translational strategy for delaying retinal degeneration and extending the therapeutic window for future restorative interventions in RP (Fig. 9). This study focused on cellular analyses of endocannabinoid-mediated effects, and future investigations will assess potential changes in FAAH activity and functional improvement in the retina of *rd10* animals.

Retinitis pigmentosa is the most prevalent form of inherited retinopathy, characterized by a progressive degeneration of photoreceptors. In mice models of RP, this neurodegeneration follows a specific temporal and spatial course within the retina, which varies depending on the model used [53]. In the *rd10* model, degeneration of the central retina begins on the 17th postnatal day, followed by the peripheral retina two days later. Photoreceptor loss under our experimental conditions is consistent with previous reports [54], which described degeneration in the central retina at P16, followed by the peripheral retina at P20. Outer segments of rod photoreceptors are shortened at the center in P10 *rd10* mice compared to WT mice [55]. This shortening occurs in the peripheral region 5 days later. This demonstrates there is a compromise in the morphological structure even before eye opening. It is still unclear what leads to this central-peripheral pattern of retinal degeneration in the *rd10*. Two hypotheses have been suggested. One possibility is a differential expression of proteins in photoreceptors located in the central retinal region, rendering these cells more susceptible to the activation of cell death mechanisms and leading to earlier degeneration. A second hypothesis relates to differences in the timing of cell differentiation, which occurs earlier in the central retina than in the peripheral retina [56]. Given these well-described differences in the temporal course of degeneration, emphasizing the importance of mapping photoreceptor degeneration in the rd10 line under each experimental condition becomes necessary.

The *rd10* model is widely used to evaluate potential drugs with neuroprotective properties [57–61]. Although there is no consensus on the age to begin treatment, many protocols have described the relationship between degeneration onset, eye opening and exposure to light. A decrease in expression of phototransduction cascade proteins was observed in animals that were maintained in the dark for a month and re-exposed to light for 24 h, with rhodopsin, transducin and guanylyl cyclase being diminished [62]. Likewise, raising mice in a dark environment slows down retinal degeneration, with fast photoreceptor death once the mice are relocated to a bright environment. It is important to note that even in *rd10* animals kept in the dark continuously for 23 or 30 days, there is an increase in oxidative stress in the retina, both in males and females [63]. Our experimental protocol considered the harmful action of light stimulus. Therefore, mice began treatment at the moment of eye opening (P13) and ended it either at P18 or at P24. This protocol is similar to that previously described [60], where treatment with AMPA and kainate receptor inhibitors in mice began at P13 and ended at P19 and P27.

Evidence supports a relationship between the endocannabinoid system and diseases of the central nervous system, including the retina. In patients, levels of AEA in the retina and the eye vary among retinopathies. AEA is increased in the cornea, ciliary body, retina and choroid of patients with diabetic retinopathy (DR) [64], however, lower levels of AEA are found in the aqueous humour of women with DR compared to healthy subjects [65]. In patients with age-related macular degeneration (AMD), there is also an increase in AEA levels in the cornea, ciliary body and choroid. While no changes were observed in the eyes of patients affected by glaucoma [66]. Few works have demonstrated the effect of cannabinoids on retinitis pigmentosa animal models [37, 41] and for the first time this study showed the involvement and neuroprotective efficacy of the endocannabinoid system in the degeneration of photoreceptors in a RP mouse model.

AEA increase can be pharmacologically induced by inhibition of FAAH, its main degrading enzyme. URB597 is one of such blockers, with an average inhibitory concentration (IC50) of 0.15 mg/kg on the brain. Intraperitoneal (i.p.) administration of URB597 0.3 mg/kg in rats acts for up to 6 h after its application, increasing brain AEA levels [67]. In addition, URB597 decreases the activity of retinal FAAH by 65% after intraperitoneal application of 0.3 mg/kg [42], evidencing the ability of URB597 to reach the retina when administered systemically. The effect of URB597 in the retina is also selective, as it increases endogenous levels of AEA but not of 2-AG [43]. Likewise, in rat brain sections, the use of 2-AG degradation inhibitors raises the levels of this endocannabinoid, while URB597 does not lead to an increase in 2-AG levels [68]. Accordingly, the *rd10* mouse model received URB597 (0.3 mg/kg, i.p.) for 6 or 12 days to inhibit FAAH. This treatment resulted in preservation of photoreceptor numbers in the peripheral retina, whereas no protective effect was observed in the central retina. In Wistar rats, i.p. administration of URB597 at the same dose increased plasma levels of AEA, palmitoylethanolamine (PEA), and oleoylethanolamine (OEA), but not 2-AG [69]. Notably, PEA has been shown to reduce the levels of inflammatory cytokines [70]. Therefore, it is likely that not only anandamide but also other endocannabinoids, such as PEA, contribute to the retinal effects observed in *rd10* animals treated with URB597. Although the effects of OEA on the retina have not yet been investigated, it represents a potentially relevant modulator that warrants further consideration.

Neuroprotection promoted by systemic FAAH inhibition in P19 *rd10* mice occurred only at the peripheral region of the retina, suggesting a regional difference in anandamide response. I.p injection might provide an uneven pharmacological distribution across different retinal regions. Tao et al. have observed very similar effects in an induced model of RP treated with Hydrogen Rich Saline administered intraperitoneally and intravitreally (i.v.). Animals that received i.p. injections had a preservation of ONL thickness at the periphery of the retina, which was not observed in the central region. In i.v. treated mice, the increase in ONL thickness was similar for both regions of the retina [71]. Another hypothesis would be a differential expression of endocannabinoid system components between retinal regions. For example, an increase in endocannabinoid levels would influence cells containing cannabinoid-responsive receptors in the periphery, but not in the center. In the retina of vervet monkeys (Chlorocebus sabaeus) there is a more intense labeling of CB1 in the GCL in the central region, as opposed to the peripheral region [72].

Intraperitoneal treatment with URB597 up to P25 indicates that FAAH inhibition induces a neuroprotective effect in the peripheral retina. These findings indicate that the treatment is still able to induce a protective effect after the peak of degeneration, despite the sharp decline in the photoreceptor population during the first three postnatal weeks in the rd10 mouse line [19, 55]. This may explain the reduced efficacy of URB597 at P25, as the rate of cell death increases from P19 onwards, potentially overriding the effects of the drug. Furthermore, daily intraperitoneal treatment may lead to desensitization of cannabinoid receptors, as CB1 receptor expression is reduced following chronic treatment with a stable anandamide analogue [32]. It would be relevant to evaluate later time points beyond P25 to assess the extent to which the treatment is able to promote photoreceptor protection. One possible explanation for why the increase in photoreceptor number is not reflected in ONL thickness at P25 may lie in the cellular reorganization that occurs following photoreceptor loss. In rd10 mice, photoreceptors at P19 are tightly packed within the ONL, whereas at P25 they are more loosely distributed, which may allow for an increased cell number without a corresponding change in ONL thickness. Supporting this observation, rd10 mice at P28 exhibit a disorganized ONL, with photoreceptors more widely spaced [19].

Increased anandamide availability can protect cells from undergoing cell death. Our data show that treatment with URB597 reduces the number of dying cells in TUNEL assays by approximately 50% at P19 and 66% at P25. Consistent with these findings, previous studies have reported that anandamide decreases the number of TUNEL-positive cells in the INL in an *in vivo* model of retinal excitotoxicity [31]. Despite the similar reduction in cell death observed at both ages, P25 animals still exhibit fewer photoreceptors than P19 animals. This may be due to incomplete photoreceptor protection at P19 following URB597 treatment, allowing continued cell loss. In addition, photoreceptors may degenerate through distinct cell death mechanisms [73]. In rd models, TUNEL labeling co-localizes with calpain and poly (ADP-ribose) polymerase (PARP), whereas cGMP and histone deacetylase (HDAC) activity do not [25].

Reactive gliosis is a process in which Müller glia undergo morphological and neurochemical changes, acquiring the ability to release molecules with homeostatic capacity that, depending on the physiological and pathological context, may also become harmful, thus playing a critical role in degenerative events [74, 75]. In the retina of the *rd10* strain, this process begins during the third postnatal week (P15) [55] and persists for approximately two months [19]. Therefore, the effect of endocannabinoid system modulation on reactive gliosis was evaluated. A hallmark of reactive gliosis is increased GFAP expression, which was analyzed by immunohistochemistry and quantified by fluorescence intensity. In *rd10* mice, no differences in GFAP fluorescence intensity were observed at P19 or P25 following subchronic treatment with URB597, indicating that inhibition of anandamide degradation did not modify reactive gliosis in the retina of these animals. Despite this, cannabinoids may influence cytokine release independently of Müller glia GFAP expression. In purified Müller glia cultures, stimulation with LPS induces reactive gliosis. Co-application of AEA or 2-AG with LPS reduces the levels of pro-inflammatory cytokines (TNF-α and IFN-γ) while increasing anti-inflammatory cytokines (IL-10 and TGF-β) after 24 h of incubation [30]. Murine Müller glia express enzymes involved in anandamide and 2-AG synthesis and degradation (NAPE and MAGL) [49], as well as CB1 and CB2 receptors, suggesting that these cells are capable of regulating retinal cannabinergic tone. Although no changes in reactive gliosis were detected following URB597 treatment, cannabinoids may still influence Müller glia expression of endocannabinoid system components and cytokine profiles. FAAH inhibition may therefore indirectly modulate the glial phenotype, shifting their molecular machinery from a pro-inflammatory to an anti-inflammatory profile.

Microglia share the same developmental origin as immune cells but reside within the central nervous system and play important roles in synaptic maintenance and preservation [76]. Iba-1 is a widely used marker for detection of activated and resting microglial cells, being expressed in cell body and cellular processes [77, 78]. In the retina of *rd10* mice, microglia proliferate and migrate to the photoreceptor layer during the second postnatal week, immediately following the rapid degeneration of photoreceptors [24, 55, 73]. This migratory behavior is also observed in patients with RP [79]. Previous studies indicate that microglia play a critical role during retinal degeneration, although it remains controversial whether they accelerate degeneration [24] or exert protective effects [80]. Microglia displays phagocytic activity, a mechanism that may underlie this dual role in protection and toxicity. Zhao et al. demonstrated that, in the *rd10* retina, microglia phagocytose more than cellular debris [73]. The effects of FAAH inhibition on microglial number and distribution were also evaluated in the *rd10* retina. No differences were observed in the number of Iba-1^+^ microglial cells following subchronic treatment, either in the ONL or across the entire retina at P19 and P25. Microglia are capable of synthesizing AEA and 2-AG, which are also involved in the regulation of microglial activity and the induction of an anti-inflammatory phenotype [81]. Specifically, 2-AG promotes microglial proliferation, whereas AEA reduces the release of pro-inflammatory cytokines [82, 83]. Murine microglia maintained in culture express CB2 receptors, and stimulation of these receptors inhibits microglial activation, reduces phagocytic activity, and decreases the release of pro-inflammatory cytokines following interferon-γ stimulation [84]. Therefore, dysregulation of components of the endocannabinoid system may influence microglial phenotype, contributing to activation and migration prior to the onset of photoreceptor degeneration. Although URB597 treatment did not alter the number of microglial cells in rd10 retinas, it is possible that FAAH inhibition modulates microglial phenotype and, consequently, cytokine production.

Oxidative stress is a major contributor to progression of photoreceptor degeneration in RP, including the *rd10* model. Proteins related to mitochondrial function are disturbed during the onset of degeneration at P18 [85], several regulators of iron homeostasis are altered [86], as well as depletion of endogenous antioxidants [87, 88]. An antioxidant response was observed in animals P19 which received URB597 intraperitoneally, with a 30% reduction in ROS levels. This might account, at least partially, to the neuroprotective effect of FAAH inhibition in *rd10* animals. Similar to our findings, URB597 has been shown to protect against ROS-induced damage in models of neurodegeneration *in vivo* [89] and *in vitro* [90]. AEA may act directly upon ROS production via mitochondrial CB1 receptors (mtCB1). Activation of mtCB1 decreases mitochondrial respiration [91], one of the main cellular ROS sources [92]. Treatment of rat brain-purified mitochondria with AEA or WIN 55212–2 limits ROS production induced by 3-Nitropropionic Acid toxicity, with the protective effect blocked by CB1 antagonists [93]. Cannabidiol (CBD) is a phytocannabinoid capable of modulating receptors, enzymes and transporters associated with the endocannabinoid system [94] and, similar to URB597, has been reported to inhibit FAAH activity and increase AEA levels [95]. Although further analyses are necessary to determine which signaling pathways are involved in the reduction of oxidative stress promoted by FAAH inhibition, CBD has been linked to activation of PKA/AMPK pathway and inhibition of NF-κB signaling under inflammatory conditions [96–98]. In the context of retinal degeneration, PI3K/Akt and MAPK pathways are important for AEA-mediated neuroprotection following i.v administration [31]. Activation of PI3K/Akt pathway promotes phosphorylation and consequent inactivation of GSK-3β. In photoreceptor cultures subjected to H_2_O_2_-induced oxidative stress, treatment with basic FGF reduced apoptosis through the PKA/GSK-3β signaling pathway [99]. *Rd10* animals at P19 naturally exhibit increased levels of inactivated GSK-3β, and daily i.p treatment with a GSK-3β inhibitor improves retinal cellular and functional responses while reducing inflammatory markers [57]. Cannabinoids have been associated with GSK-3β phosphorylation in different brain regions [100]. Oxidative stress is also associated with reduced levels of Nrf2, and its activation promotes neuroprotection in AMD, DR and RP models [101–104]. Some reports have demonstrated that GSK-3β signaling may regulate Nrf2 and NF-κB in photoreceptors, retinal pigment epithelium and Müller glia [105–107]. Collectively, these findings raise the possibility that the endocannabinoid system may attenuate neurodegeneration through activation of signaling pathways such as PI3K/Akt and/or PKA, which have GSK-3β as a downstream target. GSK-3β, in turn, may regulate transcription factors such as Nrf2 and NF-κB. Therefore, future investigations exploring the involvement of these signaling pathways may provide important insights into the mechanisms underlying cannabinoid-mediated neuroprotection in retinal degeneration.

Currently, several therapeutic interventions are being investigated to delay or prevent the harmful effects triggered by gene mutations associated with inherited retinal dystrophies. One of the major challenges for these treatments is the variability in disease onset and progression among patients. A potential therapeutic strategy to delay disease progression involves photoreceptor replacement using stem cells. However, administration of mesenchymal stem cells in RP patients did not demonstrate robust clinical outcomes after one year of follow-up [108]. Therefore, it is important to develop therapeutic approaches capable of acting on and preserving already compromised cells. Gene therapy represents an important intervention strategy, with several preclinical studies under development and some currently undergoing clinical evaluation [8]. In *rd10* mice at P4, delivery of anti-apoptotic XIAP family genes and the β-subunit of PDE increased photoreceptor survival, although did not result in significant improvement in retinal function. Thus, even with replacement of the defective gene and inhibition of apoptotic pathways during early stages of degeneration, disease progression persists [62]. Furthermore, the mutation-specific targeting required for each patient partially limits the broad applicability of gene therapy. This scenario highlights the need for treatments capable of slowing the pathological course of RP while patients await future gene repair strategies. In this context, the present pharmacological approach focused on cannabinoid modulation, aiming to demonstrate beneficial retinal effects beyond mutation-specific mechanisms.

Intraperitoneal treatment presents the advantage of enabling early drug administration, particularly considering that retinal degeneration begins during early developmental stages in several forms of RP. In rodent models, this narrow temporal window limits the applicability of i.v injections and topical ocular administration, since eye opening occurs only approximately two weeks after birth. Another strategy that may allow early intervention is nutritional supplementation during the perinatal period. Some nutraceutical compounds have been associated with endocannabinoid system modulation and beneficial retinal effects. Omega-3 and omega-6 are long-chain polyunsaturated fatty acids present in cell membranes and, similarly to endocannabinoids, act as lipid mediators with therapeutic potential in the central nervous system [109, 110]. Omega-3 supplementation modulates CB1 receptor expression, whereas nutritional deficiency alters receptor function [111, 112]. In addition, omega-3-derived compounds have been shown to activate TRPV1 receptors *in vitro* [113]. Clinical trials in RP patients supplemented with omega-3 components and/or vitamin A did not demonstrate significant improvements in visual acuity, electroretinographic responses, or optical coherence tomography parameters [114]. Therefore, these findings highlight the need to explore additional bioactive compounds. Analyses of retinal CBD bioavailability following oral administration remain poorly explored and warrant further investigation, particularly considering previous reports demonstrating broad CBD distribution across different tissues in orally treated mice [115]. Maccarone et al. demonstrated that dietary saffron supplementation attenuates light-induced retinal degeneration, potentially involving modulation of CB1 and CB2 receptors [39]. Additional studies have also reported antioxidant and anti-inflammatory effects of oral saffron administration [116–118]. A recent study investigated maternal saffron treatment starting from the 10th day of gestation and throughout the lactation period, followed by supplementation of young *rd10* mice until different key stages of degeneration. The study demonstrated that this approach promoted photoreceptor survival while improving functional, cellular, and physiological retinal parameters [119]. Therefore, such nutraceutical compounds may represent targets for combined therapeutic strategies aimed at anticipating neuroprotection during the natural course of RP. The establishment of protocols using bioactive compounds, associated or not with the endocannabinoid system, creates opportunities for investigating safe doses, systemic side effects, maternal metabolic impact, offspring development, and their effects on the visual system in hereditary retinal pathologies.

Given the reduction in oxidative stress and photoreceptor cell death observed following FAAH inhibition, modulation of the endocannabinoid system may represent a relevant component of future multi-target therapeutic strategies combining neuroprotection, antioxidant interventions, and gene-based therapies for RP.

## Data Availability

The datasets generated and analyzed during the current study are available from the corresponding author upon reasonable request.
